# Extraction and Encapsulation of Phenolic Compounds of Tunisian Rosemary (*Rosmarinus officinalis* L.) Extracts in Silk Fibroin Nanoparticles

**DOI:** 10.3390/plants10112312

**Published:** 2021-10-27

**Authors:** Kheiria Hcini, Antonio A. Lozano-Pérez, José Luis Cenis, María Quílez, Maria José Jordán

**Affiliations:** 1 Biodiversity, Biotechnology and Climate Change Laboratory (LR11ES09), Department of Life Sciences, Faculty of Science of Tunis, University of Tunis El Manar, Tunis 2092, Tunisia; 2 Department of Life Sciences, Faculty of Sciences of Gafsa, University Campus Sidi Ahmed Zarroug, University of Gafsa, Gafsa 2112, Tunisia; 3 Departamento de Biotecnología, Genómica y Mejora Vegetal, Instituto Murciano de Investigación y Desarrollo Agrario y Medioambiental (IMIDA), La Alberca, 30150 Murcia, Spain; abel@um.es (A.A.L.-P.); josel.cenis@carm.es (J.L.C.); 4 Instituto Murciano de Investigación Biosanitaria (IMIB)-Arrixaca, El Palmar, 30120 Murcia, Spain; 5 Departamento de Desarrollo Rural, Enología y Agricultura Sostenible, Instituto Murciano de Investigación y Desarrollo Agrario y Medioambiental (IMIDA), La Alberca, 30150 Murcia, Spain; maria.quilez@carm.es (M.Q.); mariaj.jordan@carm.es (M.J.J.)

**Keywords:** *Rosmarinus officinalis* L. extracts, post-distilled residues, HPLC, radical scavenging activity, DPPH, silk fibroin nanoparticles (SFNs), nanoencapsulation

## Abstract

Rosemary (*Rosmarinus officinalis* L.) is known to be an effective potential source of natural antioxidants which confer benefits to human health. Their bioactive properties are mainly due to phenolic compounds but these molecules are highly vulnerable to oxidants, light, heat, pH, water and enzymatic activities. Therefore, the stability and shelf life of phenolic compounds should be increased by being protected from chemical and physical damage by means of encapsulation prior to application. Encapsulation is becoming increasingly important in the pharmaceutical, food, cosmetics, textile, personal care, chemical, biotechnology, and medicinal industries due to its potential for stabilization and delivery of delicate and precious bioactive compounds. The aim of the present work was to describe the polyphenolic profile of Tunisian Rosemary, collected from two different bioclimatic areas, and further loading in silk fibroin nanoparticles. The loaded nanoparticles were characterized in terms of morphology, size, polydispersity, Z-potential, secondary structure of the protein, encapsulation efficiency, loading content, and antioxidant activity. On one hand, HPLC analysis revealed the presence of 18 polyphenolic compounds of whichcarnosic acid and carnosol were found to be the most abundant compounds (46.3 to 76.4 and 22.4 to 43.5 mg of compound per gram of dry plant weight (mg/g DPW) respectively), Total phenolic content (TPC) ranged from 85.8 to 137.3 mg of gallic acid equivalent (GAE)/g DPW in post-distilled rosemary extracts andantioxidant activity reached the values of 5.9 to 8.3 µmol of ascorbic acid equivalent (AAE)/g DPW). On the other hand loaded nanoparticles were almost spherical and presented nanometric size and negative Z-potential. Although the encapsulation efficiency in silk fibroin nanoparticles and the drug loading content were low in the conditions of the assay, the encapsulated polyphenols retained near 85% of the radical scavenging activity against DPPH· after 24 h. of incubation at 37 °C. The results showed that post-distilled rosemary residues had an effective potential as natural antioxidants due to their significant antioxidant activity and seemed to be useful in both pharmaceutical and food industries with beneficial properties that might confer benefits to human health and these silk fibroin nanoparticles loaded with rosemary extracts are thus a promising combination for several applications in food technology or nanomedicine.

## 1. Introduction

Rosemary (*Rosmarinus officinalis L.*) is a perennial herb that belongs to the *Lamiaceae* family, which is cultivated as medicinal plant in different areas of the world, such as the Mediterranean, Asia, and Latin America [1]. In Tunisia, this shrub is growing wild in bioclimatic zones extending from the sub-humid to the arid, with a rain fall level from 200 to 600 mm/year on sandy, calcareous or marno-calcareous soils covering an area of about 340,000 ha [2]. Leaves of rosemary have been used for a long time in Mediterranean cuisine, not only to improve or modify the flavour of foods, but also to inhibit its deterioration. Dried leaves, or their extracts, are characterized by their richness in phenolic compounds with known anti-inflammatory, antioxidant, antiaging, antibacterial, and anticancer properties [3,4]. The phenolic diterpenes, carnosic acid and carnosol, have been identified as the principal antioxidative components of these extracts [5,6]. Also, several flavonoids and phenolic compounds, such as hispidulin, cirsimaritin, apigenin, genkwanin, naringin, rutin, caffeic acid, and rosmarinic acid have been described as components of these rosemary extracts [7,8,9]. The importance of describing and quantifying these components relies on the potential biological activities that most of them exhibit. Thus, Bai et al. (2010) described the cytotoxicity, in human cancer cell lines, of some flavonoids and phenolic compounds extracted from rosemary, showing that among the fifteen components identified, carnosic acid and carnosol were the most effective as anti-proliferative agents in HL-60 cells and also they exhibited a potent anti-proliferative effects on COLO 205 cells [10].

During the last decade there has been a growing interest in the formulation of new cosmetic, food, and pharmaceutical products containing natural compounds with antioxidant activity and other beneficial properties. Unfortunately, due to their structure and nature, certain compounds such as polyphenols, are not stable and may interact easily with the matrices in which they are incorporated. Although it is crucial to benefit from the phenolic compounds, there are unsaturated bonds in the molecular structure of polyphenols and this makes them vulnerable to oxidants, exposure to light, heat, pH, water, and enzymatic activities [11]. Therefore, the stability and shelf life of phenolic compounds should be increased by being protected from chemical and physical damage prior to their application.

Encapsulation is becoming increasingly important in the pharmaceutical, food, cosmetics, textile, personal care, chemical, biotechnology, and medicinal industries due to its potential for stabilization and delivery delicate and precious bioactive compounds [12]. Thus, a bioactive compound encapsulated in a biopolymer can be efficiently protected from harmful environmental agents like light, oxygen or water [13]. Thus, encapsulation is one of the strategies used to increase the stability and shelf life of food ingredients. Encapsulation not only permits the phenolic compounds from raw vegetables or fruits to be stored, but also to be recovered under specific conditions [10,14,15,16].

Among the biomaterials that have been proposed for encapsulation in food, cosmetic, and medicinal applications, silk fibroin (SF) is a highly versatile protein utilized for centuries in the textile, and cosmetic industries. Silk is a natural polymer obtained from the cocoon of *Bombyx mori* silkworms, which is relatively inexpensive, biocompatible, biodegradable, and non-toxic FDA-approved. During the last few decades, their use has also been spread in a broad range of applications in regenerative medicine as a scaffold for tissue engineering, as well as in nanomedicine as controlled drug delivery systems [17,18]. Their extensive hydrogen bonding, amphipathic nature and high degree of crystallinity contribute to the stability of silk biomaterials [19]. Formulated as particles, silk fibroin is used in nanomedicine for its capacity to act as a reversible carrier of bioactive molecules [20,21,22]. Although SF has been investigated as a carrier for single antioxidant molecules as resveratrol [23], quercetin [24], or curcumin [25,26] among others, for volatile compounds [27], and for vegetal oils in the form of emulsions [28], to our knowledge there is only one previous example in literature of a vegetal antioxidant extract, which was obtained from olive leaves and encapsulated in silk fibroin, presented by Bayçin and coworkers [29,30].

On one hand, our study has been undertaken with the aim to identify and quantify the polyphenolic compounds of the methanolic extracts from post distilled rosemary plants (*Rosmarinus officinalis* L.), a by-product of the essential oil industry, characterizing their composition and testing their antioxidant capacity. On the other hand, we are interested in presenting the silk fibroin nanoparticles as a useful biopolymeric carrier in order to increase the stability of phenolic compounds recovered from this by-product in Tunisia. This combination being a natural product is stable, scalable and a simple method to protect the antioxidants of rosemary methanolic extracts (RME). This work constitutes a proof of concept of the revalorizing process giving a second opportunity to this valuable product, which would increase the economic value of this rain fed crop for the rural development in this country.

## 2. Results and Discussion

### 2.1. HPLC Polyphenolic Profile

Eighteen phenolics compounds were identified in the methanolic extracts of rosemary, including four phenolic acids, five phenolic diterpenes, and nine flavonoids. The results are shown in Table 1, and as expected, among the mentioned phenolic compounds, carnosic acid and carnosol were the major diterpenic components quantified in rosemary extract followed by rosmarinic acid, hesperidin and 12-me-carnosic acid, since previous studies conducted by Hcini et al. (2013) showed similar results regarding the major polyphenolic profile of rosemary plants harvested from populations located at three different geographic origins (Beja, Sidi Bouzid and Gabes) in Tunisia [31].

In the present study locations belonging to two different bioclimatic regions were prospected, showing, as a general pattern, that the differences in polyphenolic content should be attributed more to the genetic inheritance of the plants, than to the area of prospection. For instance, as observed in Table 1, rosmarinic acid, phenolic acid known as an important contributor of the antioxidant capacity of the rosemary extract [32], was detected at the highest concentration in plants prospected in the Elkef–Sers (KS) populations, showing statistically significant differences (*p* < 0.05) with respect to those plants harvested in closer areas such as ElKef–Menzel Salem (KMS) and being equal to those prospected at Gafsa–Sened (GS) location, where the climatic conditions correspond to an arid lower zone.

However, when the diterpenic fraction is analysed this pattern changes, thus, carnosic acid, major diterpene identified and precursor of the rest of diterpenes (carnosic acid’s oxidation derivative products) quantified in these extracts, was detected at the highest concentration in plants harvested at the Gafsa–Orbata (GO) population (*p* < 0.05), located in an arid low bioclimatic area, where the climatic conditions are the most extreme among the areas under study. This behaviour was also detected for salvianolic acid A, since also for these components high temperatures and low rainfall also increase the presence of this polyphenol in rosemary leaves for these components.

Thus, as a main conclusion, not all the components that define the major polyphenolic profile of rosemary leave extracts are affected at the same level by the bioclimatic conditions in which the plant grows. Just salvianolic acid A, and the diterpenic fraction increase their concentrations in plants located at low arid zones with hard climatic conditions.

In contrast to this, studies by Yeddes et al. (2019) on the effect of bioclimatic area and season on phenolics and antioxidant activities of rosemary growing wild in Tunisia showed that there was a strong correlation between antioxidant activity and phenolic content depending on bioclimatic and seasonal effects [33]. However, our results are in agreement with those published previously by Luis et al. (2007) and Jordán et al. (2013) [34,35], that the effect of abiotic stress on rosemary antioxidant compounds revealed that stressed plants produced increased concentrations of caffeic and carnosic acids, while other secondary metabolites, such as rosmarinic acid, naringenin, cirsimaritin, hispidulin and carnosol, showed different responses depending on the plant analysed.

### 2.2. Total Phenolic Content and Antioxidant Activity

Following the characterization of the rosemary methanolic extracts, the study of the antioxidant activity was carried out by measuring the total phenolic content and the radical scavenging activity against DPPH^•^. Results, shown in Table 2, revealed that in agreement with the previous major polyphenolic profile quantified, plants harvested at the GO population followed by the KS area showed the highest antioxidant capacities measured in both in vitro tests. In this way, the presence of the phenolic diterpenes and salvianolic acid A at the highest concentration in GO plants and rosmarinic acid in KS would explain these high antioxidant capacities. Several authors have published the important role of carnosic acid, carnosol, rosmadial, and rosmarinic acid on the antioxidant power of the rosemary extract including Tsimogiannis et al. (2016); Loussouarn et al. (2017) and Li et al. (2018), among others [36,37,38].

The high biological activities exhibited by these components, particularly rosmarinic acid and carnosic acid have led to the publications of several studies related to the potent antioxidant power of these phenolic compounds. In this sense, Chkhikvishvili et al. (2013) published their protective effect from the oxidative stress, induced by hydrogen peroxide, evaluated in Jurkat cells [39]. Later Andrade et al. (2018) reported the protective role of rosemary extract in preventing colds, rheumatism, and pain of muscles [40], and more recently Pérez-Sánchez et al. (2019) reposted the antitumor activity through their capacity to inhibit various signatures of cancer progression and metastasis [41]. These studies, among many others, justify the importance of phenolic acids for preventing or even curing some diseases. On the basis of these statements, and considering the importance of stabilizing these active compounds through the encapsulation, as it was stated at the introduction section, three rosemary extracts, among the plants under study, were selected on the basis of their richness in diterpenes and rosmarinic acid, to accomplish the encapsulation of methanolic extracts in silk fibroin nanoparticles.

### 2.3. Characterization of Rosemary Methanolic Extracts Loaded in Silk Fibroin Nanoparticles (RME–SFNs)

**Figure 1:**
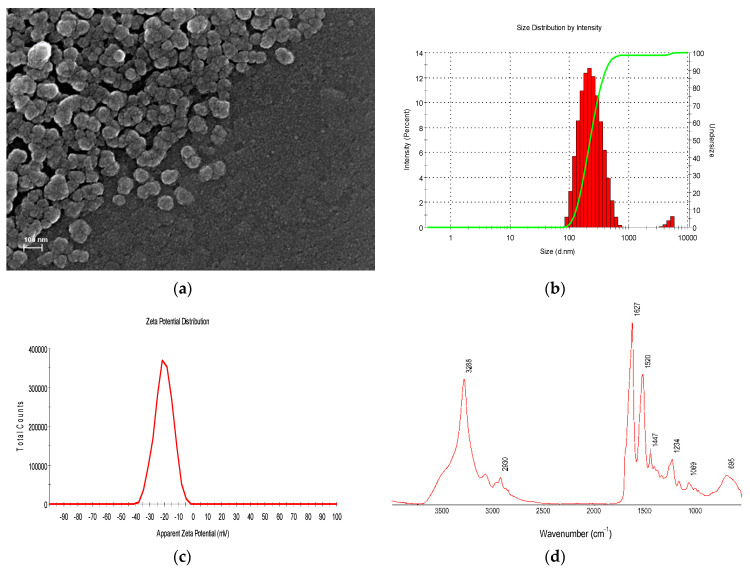
Characterization of RME–SFNs: (**a**) FESEM image of the freeze dried nanoparticles. (**b**) Size distribution (diameter in nm) by Intensity (%) measured by DLS. (**c**) Z-potential distribution and (**d**) ATR–FTIR spectrum.

The RME–SFNs nanoparticles obtained from the desolvation of the silk fibroin peptides in the selected *rosemary* methanolic extracts presented a pale green color and after freeze drying that could be easily dispersed in water, forming an homogenous suspension. Although is stable for several weeks at 4 °C without change in color, that presented slow particle aggregation and trend to sedimentation after 24–48 h. Field Emission Scanning Electron Microscopy (FESEM) images of the freeze dried RME–SFNs (Figure 1a) revealed a pseudo-spherical morphology of about 150 nm in size which was slightly smaller than the hydrodynamic size obtained by Dynamic Light Scattering (DLS) measurements (Figure 1b) but similar to those previously described for equivalent unloaded nanoparticles [23,25,42,43]. The aggregation observed for the lyophilized RME-SFNs was higher than that for SFNs, due the effect of the polyphenols adsorbed on the surface, in agreement with the quercetin-loaded nanoparticles previously presented by our group [23].

The hydrodynamic properties and stability of the particles in aqueous suspension were tested by using dynamic light scattering (DLS). The DLS measurements also showed the increase of the size of the nanoparticles when compared with the SFN control formed in the absence of the RME. The size distribution of the aqueous suspension of the RME–SFNs with a nanometric Z_average_ of 217.4 ± 5.0 nm, are detailed in Table 3. All samples showed a moderately negative Z-potential (ζ) with a narrow distribution of the apparent Zeta Potentiel of −17.4 ± 2.8 mV as is shown in Figure 1c, from the −36.9 ± 3.8 mV for the unloaded SFNs, that agrees with the aggregation phenomenon of the aqueous suspensions of loaded nanoparticles due to mild electrostatic repulsion forces among particles moderatly charged. This “shielding effect” of the polyphenols, equivalent to the “protein corona” on the nanoparticle surface, which have been discussed previously in literature, might explain this effect [44].

An example of infrared spectrum of the loaded nanoparticles is shown in Figure 1d. The peak profile of the RME–SFNs is similar to that described in literature for unloaded SFNs [23,45,46]. According to that, the assignation of the main “vibrational bands” was performed resulting in the identification of a strong peak at 1626 cm^−1^ which was assigned to the C=O stretching of Amide I region and a second strong peak at 1524 cm^–1^, corresponding to the N–H bending of Amide II. This peak profile is characteristic of the crystalline and insoluble β-sheet structure [47]. The characteristic signals of the methanolic extracts were partially masked in the recorded spectrum of RME–SFNs by the intense signals of the SF.

### 2.4. Determination of the Polyphenolic Loading Content (PLC) and Encapsulation Efficiency (EE%)

The average values of PLC and the EE% of the selected RME in the SFNs are shown in the Table 4. As can be observed, the encapsulation efficiency of the extracted rosemary components in the SFNs depends on the chemical behaviour of each polyphenol and their affinity to the surface of silk nanoparticle. Diterpenes, with nonpolar nature seem to be the components retained at the highest concentrations with the lactone carnosol being the most highly retained. Carnosic acid, a major component quantified in the extract was not detected at the SFNs, this diterpene is characterized by its high antioxidant power, and as described by Zhang et al. (2012) [48], in a mixture of three polyphenols (carnosic acid, carnosol, and rosmarinic acid) carnosic acid serves to maintain levels of carnosol, though it does so at least in part at the cost of its own degradation. This affirmation would explain why carnosic acid was not detected in any of the three nanoencapsulation assays. The phenolic acids salvianic and rosmarinic acids were also retained as major components in the SFN, and as occurs with diterpenes, not a higher concentration in the rosemary extract implies a bigger retention in the SFNs. This situation can be justified by to the drug loading capacity of these nanoparticles. Although, further experiments are needed in order to justify this phenomenon.

### 2.5. DPPH· Scavenging Activity of the Silk Fibroin Nanoparticles Loaded with Phenolic Compounds

After the determination of the composition of the polyphenols loaded in the nanoparticles we tested the radical scavenging activity (RSA) against DPPH· and the stability along the time of incubation at 37 °C of the original RME, the RME loaded into SFNs and the free SFNs as control. Results presented in Table 5 showed that although nanoparticles abtained by co-precipitation method contain only small amounts of the initial RSA of the extract, only the 1.05 ± 0.05%, about 84% of the initial RSA preserved at the end of the incubation time (24 h), when polyphenols were loaded in the nanoparticles. In contrast, the RSA of the free extract dramatically decreased along the incubation time. Thus, a decreasing of about 25% of the RSA after 6 h and a further decreasing to less than 49% of the initial RSA after 24 h of incubation. This effect has been previously described for silk fibroin nanoparticles due the strong interactions between the phenolic compounds and the silk fibroin [23,24,25]. The unloaded nanoparticles used as control showed also a low radical scavenging activity probably due the presence of tyrosine residues in the silk fibroin sequence.

In conclusion, the DPPH·-scavenging activity assay after 24 h. Of incubation at 37 °C confirmed that the RSA of the RME loaded into the SFNs were better preserved in comparison with the free RME.

## 3. Materials and Methods

### 3.1. Collection of Plant Materials

In the present study 40 individual rosemary shrubs were randomly collected in the spring of 2017 from four wild populations located at two different bioclimatic areas (semi-arid superior and lower arid) at the full bloom phenological stage. Voucher specimens of rosemary from every location are deposited at the Herbarium of Departamento de Desarrollo Rural, Enología y Agricultura Sostenible, Instituto Murciano de Investigación y Desarrollo Agrario y Medioambiental (IMIDA). La Alberca (Murcia, Spain).

Details of collection sites are given in Table 6. Fresh aerial parts of individual plants were firstly dried at room temperature for ten days and afterwards dried in a forced-air drier at 35 °C for 48 h, until they reached a constant weight.

### 3.2. Chemicals and Reagents

Silk cocoons (SC) were obtained from silkworms *Bombyx mori* reared in the sericulture facilities of IMIDA (Murcia, Spain) and raised on a diet of fresh natural *Morus alba* L. leaves. Silk cocoons were processed following previously described procedures [49]. All reagents and solvents were purchased from Sigma-Aldrich (Madrid, Spain) with the exception of methanol (Honeywell, Germany).

### 3.3. Preparation of the Plant Extracts

In order to avoid interferences from the essential oil components, individual plants were firstly distilled in a Clevenger system, after this, the oil free distilled plant material was dried in a forced-air drier at 35 °C for 48 h (until it reached a constant weight) and then ground to pass through a 2-mm mesh. Dried samples (0.5 g) were extracted using 150 mL of methanol in a Soxhlet extractor (B-811) (Buchi, Flawil, Switzerland), for 2 h under a nitrogen atmosphere. Methanolic extracts (ME) were taken to dryness at 35 °C under vacuum conditions in an evaporator system (SyncorePolyvap R-96) (Buchi, Flawil, Switzerland). The residue was re-dissolved in methanol and made up to 5 mL [50]. The yield of the extracts was expressed in terms of milligrams of dry methanolic extract per gram of dry plant weight. Final extracts were kept in vials at −80 °C until their corresponding analyses.

### 3.4. Characterization of the Plant Extracts

The methanolic extracts of 40 individual samples of wild rosemary shrubs from two different bioclimatic areas of Tunisia were characterized by means of HPLC analysis, total phenolic contents, and radical scavenging activity against the DPPH.

#### 3.4.1. HPLC Analysis

For the HPLC analysis, a method adapted from Zheng and Wang [7], was performed on a reverse phase ZORBAX SB-C18 column (4.6 × 250 mm^2^, 5 μm pore size, Agilent Technologies, USA) using a guard column (ZORBAX SB-C18 4.6 × 12.5 mm^2^, 5 μm pore size, Agilent Technologies, USA) at ambient temperature. Extracts were passed through a 0.45 μm filter (Millipore SAS, Molsheim, France) and 20 μL were injected in an Agilent 1200 (Germany) system equipped with a G1311A binary pump and G1315A photodiode array UV/Vis detector. The mobile phase was acidified water containing 0.05% formic acid (A) and acetonitrile (B). The gradient was as follows: 0 min, 5% B; 10 min, 15% B; 30 min, 25% B; 35 min, 30% B; 50 min, 55% B; 55 min, 90% B; 57 min, 100% B and then held for 10 min before returning to the initial conditions. The flow rate was 1.0 mL/min and the wavelengths of detection were set at 280 and 330 nm. Identification of the phenolic components was made by comparison of retention times and spectra with those of commercially available standard compounds. For quantification, linear regression models were determined using standard dilution techniques. Phenolic compound contents were expressed in milligram per gram of dry plant weight (mg compound/g DPW).

#### 3.4.2. Determination of Total Phenolic Content

Total phenolic content in the extracts was determined by the Folin-Ciocalteu method [51]. Briefly, 15 μL of methanolic extracts were added to 1185 μL of distilled water and 75 μL of Folin-Ciocalteu reagent. A vigorous stirring was performed and 225 μL of a sodium carbonate solution (20%) were added. After 30 min of incubation, the absorbance of the resulting blue-colored solution was measured at 765 nm and 25 °C with a Shimadzu (UV-2401PC, Japan) spectrophotometer. Standard curve was prepared by using different concentrations ranging from 100 to 1000 mg/L of gallic acid (GA). Total phenolic content was expressed as mg gallic acid equivalent/g of dry plant weight (mg GAE/g DPW). Analyses were done in triplicate.

#### 3.4.3. DPPH· Scavenging Activity

Radical Scavenging Activity (RSA) was analysed following the method described by Yen and Duh with some modifications [52]. Briefly, 50 μL of methanolic extract were added to eppendorf tubes containing 850 μL of methanol, and then 100 μL of DPPH· 1 mM in methanol. After 30 min of reaction at 25 °C and protected from light, the scavenging activities of the samples and standards (ascorbic acid, 10–500 µM in methanol) were evaluated by measuring the absorbance at 515 nm, in a Synergy MX UV–Vis spectrometer (BioTek Instruments Inc., Winooski, VT, USA). For each sample concentration tested, the inhibition percentage (%I) of DPPH· in the steady state was determined following the Equation (1):

%I = [(Abs_blank_ − Abs_sample_)/Abs_blank_] × 100 (1)

Results were expressed as μmol of ascorbic acid equivalents per gram of dry plant weight (μmol AAE/g DPW).

### 3.5. Preparation of Silk Fibroin Aqueous Solution (SF)

Silk fibroin solutions were prepared according to the method previously described by our group without modifications [23,53]. Briefly silk cocoons were chopped in small pieces, degummed with Na_2_CO_3_ 0.02N for 120 min in order to remove the soluble sericins and obtain smaller nanoparticles [53], washed with distilled water and left to dry for 24 h. Then the silk fibroin fibers were dissolved in Ajisawa’s solvent system [54], composed by a mixture of CaCl_2_/ethanol/H_2_O (1:2:8 in molar ratio) for 3 h at 65 °C. Once, dissolved the silk solution was dialyzed for 48 h against ultra-pure water using a cellulose membrane (cut-off 3.5 KD) and the resultant solution was centrifuged at 6000× *g* for 10 min and 8 °C (Eppendorf Centrifuge 5810R equipped with a rotor F-34-6-38) in order to remove protein aggregates or impurities and stored at 4 °C until use.

### 3.6. Preparation of Silk Fibroin Nanoparticles Loaded with Phenolic Compounds

In order to accomplish this assay, three rosemary extract were selected from the GO population, on the basis of their high polyphenolic content and antioxidant capacity. The loading into the silk fibroin nanoparticles (SFNs) was carried out by a coprecipitation method, following the protocol described by Montalbán et al. with slight modifications [25]. Briefly, the freshly prepared SF aqueous solution was slowly dripped into vigorously stirred methanolic rosemary extract as the coagulant solvent. After a few drops, a cloudy suspension appeared and the suspension was stirred for 2 h in order to complete the formation of the β-sheet secondary structures of the silk fibroin and the loading of phenolic compounds onto the nanoparticles. Then, the particles were recovered by centrifugation at 12,000× *g* for 15 min at 8 °C (Eppendorf Centrifuge 5810R equipped with a rotor F-34-6-38).

The supernatant was decanted and the pelleted nanoparticles were washed with ultrapure water (3 cycles of resuspension in water with vortex, ultrasonication and centrifugation). After lyophilizing the particles for 72 h at −55 °C and 0.5 mbar (Edwards Modulyo 4K Freeze Dryer), the rosemary methanolic extract loaded nanoparticles (RME-SFNs) were obtained in the form of a dry powder. Empty nanoparticles (SFNs) as controls were prepared by using methanol as solvent instead of the methanolic extracts.

### 3.7. Characterization of Rosemary Methanolic Extract Loaded in Silk Fibroin Nanoparticles (RME–SFNs)

The characterization of the nanoparticles was performed using common techniques such as Field Emission Scanning Electron Microscopy (FESEM), Dynamic Light Scattering (DLS), and Attenuated Total Reflectance-Fourier Transformed Infrared Spectroscopy (ATR-FTIR). The nanoparticles were observed by FESEM using a MERLINTM VP COMPACT (Carl Zeiss Microscopy S.L., Oberkochen, Germany). An aliquot (50 μL) of an aqueous suspension of nanoparticles was dropped onto a clean glass wafer before drying overnight and gold-coated. The morphology was studied in images at different magnifications using an SE2 detector (accelerating voltage of 15 kV, WD = 9.3 mm and aperture size = 30.00 mm). The size distribution of the particles, including mean diameter (Z-average), Polydispersity (PdI) and Zeta potential (ζ), were measured using a Malvern Zetasizer Nano ZSP (Malvern Instruments Ltd., Malvern, UK). The Z-average diameter and ζ values were calculated with the Zetasizer software V. 7.14 provided by the manufacturer. All measurements were performed in quintuplicate (12 runs/measurement) in purified water at 25 °C and values expressed as mean ± SD. Attenuated Total Reflectance Fourier Transformed Infrared Spectroscopy (ATR–FTIR) analysis was performed in order to detect the possible structural changes of SF after loading with the phenolic compounds. Each spectrum was acquired on a Nicolet iS5 spectrometer, equipped with an iD5 ATR accessory (Thermo Scientific, USA) controlled by OMNIC Software Ver. 6.1.0.0038. Measurements were made in absorbance mode with a resolution of 4 cm^−1^, a spectral range of 4000–550 cm^−1^, in 64 scans, using N-B strong apodization and Mertz phase correction. The analysis focused on the 1700–1400 cm^−1^ range, which provides most information on the FTIR spectra of SF.

### 3.8. Determination of the Polyphenolic Loading Content (PLC) and Encapsulation Efficiency (EE%)

The PLC and EE % of the methanolic extracts in the SFNs were determined by HPLC analysis following the previously described protocol in Section 3.4.1 with slight modifications [55]. Each sample of freeze-dried powder of RME–SFNs was weighted (~100 mg) in an eppendorf tube and extracted with 1 mL of methanol by dispersion with vortex and sonication for 4 min. After this, the mixture was centrifuged at 12,000× *g* for ten minutes and the supernatant was aspired and used for analysis. The extraction was conducted twice and both supernatants were mixture and made up to 2 mL in a volumetric flask. The methanolic extracts were analysed by HPLC and the phenolic compound contents were expressed in microgram of polyphenol per milligram of dry rosemary methanolic extract loaded nanoparticles (µg/mg RME–SFNs). Preparations were performed in triplicate. The EE% was obtained using the Equation (2).

(EE%) = (Weight RME in nanoparticles/Weight RME in loading solution) × 100(2)

### 3.9. DPPH· Scavenging Activity of the Silk Fibroin Nanoparticles Loaded with Phenolic Compounds

Radical-scavenging activity was analysed following the method previously described in Section 3.4.3, with some modifications. In this case, 100 μL of sample (methanolic suspension of RME–SFNs, 10 mg/mL) were added to vials containing 800 μL of methanol, and finally 100 μL of DPPH· 1 mM in methanol were added to initiate the reaction. Samples were centrifuged prior measurements in order to eliminate the interference of the nanoparticles. Results were expressed as μmol of ascorbic acid equivalents per milligram of dry powder of loaded nanoparticles (μmol AAE/mg RME-SFNs). In order to determine whether the scavenging activity of the extract is retained after loading in SFNs, the radical scavenging activity (RSA) was measured along the incubation at 37.0 ± 0.2 °C at 6 h and 24 h and compared with the initial RSA. Aqueous suspensions of free RME at 0.5 mg/mL or the loaded nanoparticles at 10mg/mL were prepared in Eppendorf tubes and incubated in water Nüve NB20 bath (NÜVE SANAY, Ankara, Turkey). At the predetermined time of incubation, 100 μL of each suspension were taken for DPPH· assay, diluted with methanol and mixed with 100 μL of DPPH 1 mM, as described in the previous paragraph.

### 3.10. Statistical Analyses

All experiments were performed in triplicate (*n* = 3) and data were reported as means ± standard deviation (SD). A one-way ANOVA was carried out to assess for significant differences (significant model was accepted for a *p*-value < 0.05) using the IBM SPSS Statistic Program (v. 25). Next, Fisher’s LSD pairwise comparison was performed on the data.

## 4. Conclusions

Overall, the major polyphenolic profile of methanolic extract from post distilled rosemary leaves revealed, as expected, the presence of the diterpenes carnosic acid and its derivatives carnosol and 12-me-carnosic as major components, along with rosmarinic acid and hesperidin. Rosemary residues have proven to be an effective potential source of polyphenols and could be useful in replacing or even decreasing synthetic antioxidants in foods, cosmetics and pharmaceutical products. The positive correlation between the phenolic content and the antioxidant capacity confirmed that the phenolic constituents are responsible for the antioxidant activity of rosemary. This knowledge allowed for the selection of three individual plant extracts for the development of the nanoencapsulation assays. The encapsulation of the polyphenols from the rosemary methanolic extracts in silk fibroin nanoparticles increased their size to ~220 nm and shifted their ζ to -17 mV, still useful for cell penetration. Although the described method of encapsulation presents a low encapsulation efficiency and drug loading content, interestingly the adsorption onto silk protects the polyphenols against the degradation. In reference to the loading polyphenol content determinations, diterpene fraction along with the phenolic acids salvianic and rosmarinic acids were retained as major components in the SFNs and although a deeper study should be developed, an initial approach indicated that a higher concentration of the rosemary extract does not always imply greater retention in the SFNs. Thus, further experiments are needed in order to improve the loading efficiency of the aromatic plants extracts nanoencapsulation.

## Figures and Tables

**Table 1:** HPLC polyphenolic profiles of *R. officinalis* L. methanolic extracts from the different bioclimatic areas.

	KS	KMS	GO	GS
**Phenolic acids**				
Salvianic Acid	1.10 ± 0.13 ^ab^	1.13 ± 0.23 ^ab^	1.21 ± 0.39 ^b^	0.84 ± 0.13 ^a^
Caffeic Acid	1.00 ± 0.22 ^b^	0.90 ± 0.11b ^ab^	0.74 ± 0.26b ^ab^	0.69 ± 0.27 ^a^
Rosmarinic Acid	29.91 ± 9.33 ^c^	17.96 ± 3.25 ^ab^	26.02 ± 5.88 ^bc^	16.77 ± 7.59 ^a^
Salvianolic Acid A	1.76 ± 0.44 ^a^	1.20 ± 0.34 ^a^	2.62 ± 0.84 ^b^	1.32 ± 0.40 ^a^
**Flavonoids**				
Luteolin -7-O-Rutinoxide	0.98 ± 0.40 ^b^	0.74 ± 0.18 ^ab^	0.74 ± 0.37 ^ab^	0.57 ± 0.25 ^a^
Luteolin-7-Glucoronide	2.56 ± 0.80 ^b^	1.89 ± 0.59 ^ab^	1.15 ± 0.49 ^a^	1.28 ± 0.62 ^ab^
Hesperidin	10.41 ± 2.79 ^a^	14.0 ± 2.69 ^b^	10.6 ± 2.77 ^ab^	9.85 ± 3.47 ^a^
Luteolin	0.77 ± 0.10 ^a^	0.97 ± 0.13 ^b^	0.81 ± 0.11 ^ab^	0.78 ± 0.18 ^a^
Apigenin	0.23 ± 0.05 ^a^	0.24 ± 0.06 ^a^	0.24 ± 0.05 ^a^	0.2 ± 0.05 ^a^
Hispidulin	0.34 ± 0.05 ^a^	0.48 ± 0.09 ^b^	0.41 ± 0.09 ^ab^	0.37 ± 0.08 ^a^
Cirsimaritin	1.21 ± 0.58 ^a^	1.53 ±0.48 ^a^	1.32 ±0.41 ^a^	1.16 ± 0.42 ^a^
Genkwanin	3.30 ± 1.33 ^a^	2.51 ± 0.71 ^a^	2.07 ± 0.84 ^a^	2.05 ± 1.22 ^a^
Salvigenin	1.05 ± 0.31 ^a^	1.13 ± 0.3 ^a^	1.59 ± 0.5 ^b^	1.22 ± 0.37 ^ab^
**Diterpenes**				
Rosmadial	1.83 ± 0.32 ^a^	2.49 ± 0.41 ^bc^	2.68 ± 0.54 ^c^	1.98 ± 057 ^ab^
7-CH_3_-Rosmanol	1.45 ± 0.35 ^a^	1.49 ± 0.26 ^a^	3.73 ± 0.75 ^b^	1.12 ± 0.31 ^a^
Carnosol	26.94 ± 4.86 ^ab^	29.95 ± 3.57 ^b^	43.53 ± 4.18 ^c^	22.36 ± 4.17 ^a^
Carnosic	57.33 ± 22.37 ^a^	54.74 ± 9.24 ^a^	76.36 ± 12.87 ^b^	46.27 ± 12.01 ^a^
12-CH_3_- Carnosic acid	9.60 ± 4.86 ^a^	10.60 ± 4.00 ^a^	16.70 ± 5.84 ^b^	10.6 ± 2.78 ^a^

Code: ElKef–Menzel Salem (**KMS**), Elkef–Sers (**KS**), Gafsa–Orbata (**GO**) and Gafsa–Sened (**GS**). Contents of phenolic compounds expressed as mg compound/g of dry plant weight (mg of compound/g DPW). Results are expressed as means ± standard deviation (*n* = 10). The different lower-case letters (a–c) in the same column indicate significantly-different values (*p* < 0.05).

**Table 2:** Total phenolic content (TPC) and Radical Scavenging Activity (RSA,) of methanolic rosemary extracts from the different bioclimatic areas.

	Superior Semi-Arid		Lower Arid	
	KS	KMS	GO	GS
TPC (mg GAE/g DPW)	109.1 ± 23.1 ^b^	85.8 ± 28.4 ^a^	137.3 ± 15.6 ^c^	87.8 ± 15.0 ^a^
RSA (μmol AAE/g DPW)	7.5 ± 1.3 ^b^	6.8 ± 1.2 ^a^	8.3 ± 1.3 ^c^	5.9 ± 1.3 ^a^

Code: ElKef–Menzel Salem (**KMS**), Elkef–Sers (**KS**), Gafsa–Orbata (**GO**) and Gafsa–Sened (**GS**). Results are expressed as means ± standard deviation (*n* = 10). The different lower-case letters (a–c) in the same row indicate significantly-different values (*p* < 0.05).

**Table 3:** Characterization of the silk fibroin nanoparticles by Dynamic Light Scattering.

Sample	Z_average_ (d.nm)	Polydispersity	Z_potential_ (mV)
Control SFNs	148.0 ± 1.6	0.089 ± 0.029	−36.9 ± 3.8
RME-SFNs	217.4 ± 5.0	0.185 ± 0.018	−17.4 ± 2.8

Values expressed as mean ± standard deviation (*n* = 3).

**Table 4:** Polyphenolic loading (PLC, μg compound/mg RME-SFNs) and Encapsulation efficiency (EE, %) of the selected Rosemary extracts in RME–SFNs.

	PL	EE%
**Phenolic acids**	**0.096 ± 0.011**	**0.055 ± 0.003**
Salvianic Acid	0.010 ± 0.006	0.065 ± 0.020
Caffeic Acid	0.005 ± 0.002	0.103 ± 0.034
Rosmarinic Acid	0.080 ± 0.007	0.058 ± 0.005
Salvianolic Acid A	N.D.	N.D.
**Flavonoids**	**0.163 ± 0.011**	**0.154 ± 0.031**
Luteolin-7-O-Rutinoxide	0.026 ± 0.004	0.629 ± 0.135
Luteolin-7-Glucoronide	0.023 ± 0.004	0.254 ± 0.045
Hesperidin	0.025 ± 0.007	0.051 ± 0.024
Luteolin	N.D.	N.D.
Apigenin	0.007 ± 0.001	0.621 ± 0.094
Hispidulin	0.014 ± 0.002	0.514 ± 0.098
Cirsimaritin	0.023 ± 0.003	0.233 ± 0.021
Genkwanin	0.024 ± 0.003	0.211 ± 0.029
Salvigenin	0.021 ± 0.002	0.187 ± 0.003
**Diterpenes**	**0.265 ± 0.037**	**0.026 ± 0.001**
Rosmadial	0.009 ± 0.002	0.031 ± 0.006
7-me-Rosmanol	0.105 ± 0.019	0.199 ± 0.071
Carnosol	0.150 ± 0.050	0.052 ± 0.011
Carnosic acid	N.D.	N.D.
12-me- Carnosic acid	N.D.	N.D.
**TOTAL**	**0.523 ± 0.052**	**0.041 ± 0.004**

Results are expressed as means (*n* = 3) ± standard deviation.

**Table 5:** Evolution of the Radical Scavenging Activity (RSA) determined by DPPH· Assay after the incubation period of the aqueous suspensions at 37 °C. Values are expressed in μmol AAE/mg of the samples.

	RSA (μmol AAE/mg)		
Sample	t = 0 h	t = 6 h	t = 24 h
RME	13.32 ± 1.15	9.99 ± 0.52	6.37 ± 0.31
RME–SFNs	0.18 ± 0.01	0.17 ± 0.01	0.15 ± 0.01
SFNs	0.04 ± 0.01	0.03 ± 0.01	0.05 ± 0.01

RME: Rosmary Methanolic Extracts; RME–SFNs: Rosmary Methanolic Extract loaded in Silk Fibroin Nanoparticles: SFNs: Unloaded Silk Fibroin Nanoparticles used as Control. Values expressed as mean± standard deviation (*n* = 3).

**Table 6:** Samples collection sites and their eco-geographic characteristics. Bioclimatic Map of Tunisia According to the Classification of Emberger, published by the Tunisian Republic. National Institute of Forest Research.

Collection Site *	Bioclimatic Stage	Rainfall (mm/year)	Average Temp (°C)	Geographical Location		
				Longitude (N)	Latitude (E)	Altitude (m)
ElKef–Menzel Salem (KMS)	Superior semi-arid	446	16.2	35°51′24.0″	8°28′34.0″	995
Elkef–Sers (KS)	Superior semi-arid	441	16.9	36°4′36.2″	9°1′21.8″	887
Gafsa–Orbata (GO)	Lower Arid	223	19.6	34°22′49.8″	9°3′23.4″	1165
Gafsa–Sened (GS)	Lower Arid	222	17.3	34°28′1.2″	9°16′1.2″	431

* N = 10 Plants.
